# Exogestation for treating premature births and congenital diseases: Artificial womb technology edges towards first trials in humans

**DOI:** 10.1038/s44319-023-00022-4

**Published:** 2024-01-05

**Authors:** Philip Hunter

**Affiliations:** Freelance Journalist, London, UK

**Keywords:** Development, Economics, Law & Politics, Methods & Resources

## Abstract

Exogestation has made considerable advances lately to the point that first therapeutic applications in human can be considered.

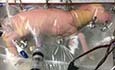

The successful demonstration of Artificial Womb Technology (AWT) in animal models has heightened interest in human applications, but with significant technological, ethical and legal challenges. The first clinical trials will involve cases of premature birth at 22 to 24 weeks when neonatal Intensive Care Unita (ICU) is unviable. Eventually, there are hopes of applying the same technology to babies identified with congenital defects that can only be effectively treated through surgical or other interventions early in pregnancy.

“... there are hopes of applying the same technology to babies identified with congenital defects that can only be effectively treated through surgical or other interventions early in pregnancy.”

Otherwise known as ectogestation, AWT involves transfer of a foetus to an external womb surrogate filled with a fluid containing electrolytes. During transfer the placenta must quickly be reattached to an external supply for nutrient delivery, removal of waste products, and exchange of oxygen and CO_2_. This involves the critical stage of intrauterine to extrauterine transplantation, which must avoid damage to the foetus, while avoiding harm to the mother.

## First demonstration of viability

AWT has been subject to research in animal models for some years, largely because of its potential application to humans. A major step forward came in 2017 when scientists at the Children’s Hospital of Philadelphia (CHOP) published a successful demonstration of ectogestation of a premature lamb foetus (Partridge et al, 2017).

Previous attempts to extend gestation in extracorporeal systems outside the womb had only limited success, usually with complications ensuing. The 2017 study though made significant progress, while also demonstrating an approach that might help make the transition to humans where the foetus is relatively smaller at the same developmental stage. Lambs, like most mammals, are born larger relative to adult size and more physically developed than human babies.

“Previous attempts to extend gestation in extracorporeal systems outside the womb had only limited success, usually with complications ensuing.”

**Figure 1:**
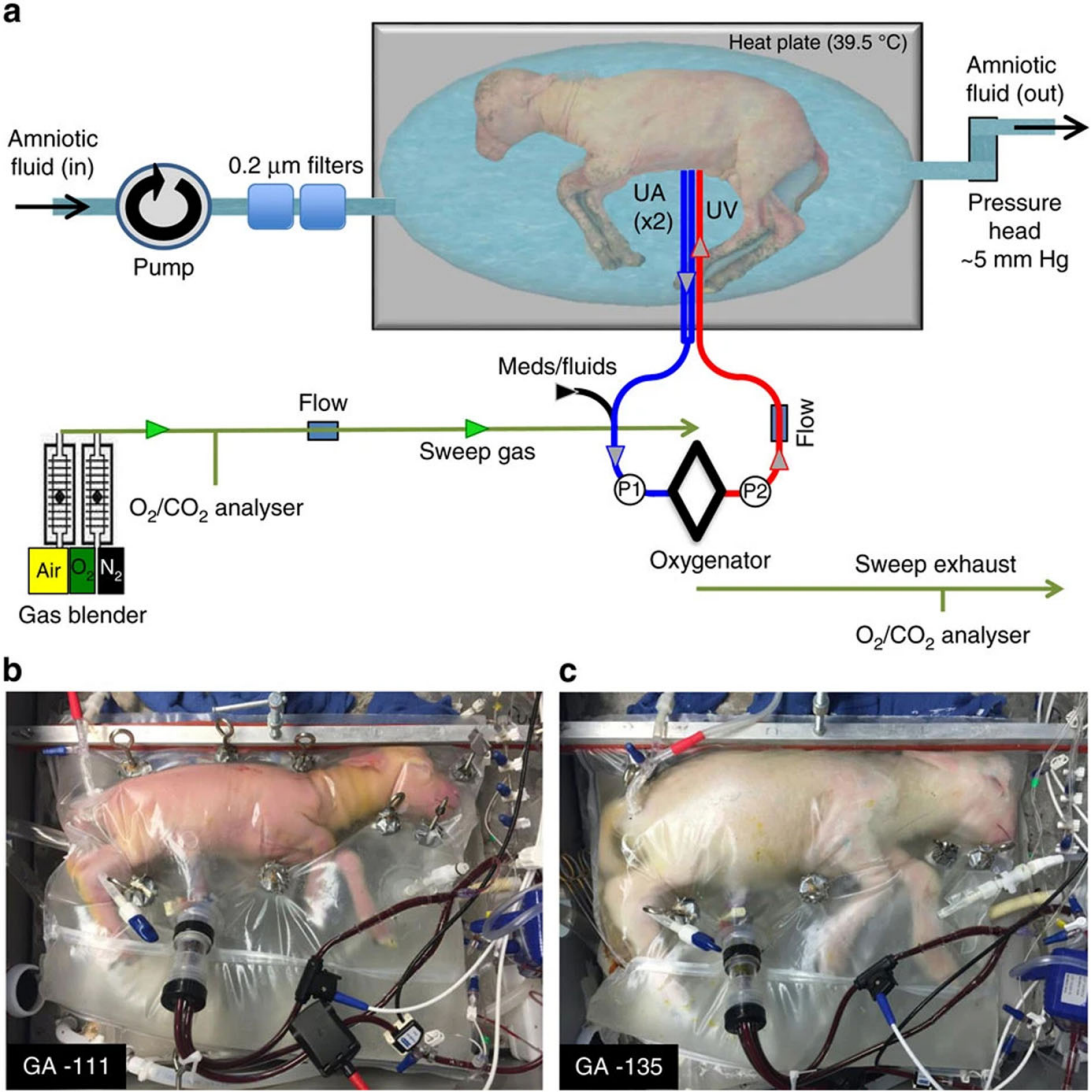
The artificial womb system at Children’s Hospital of Philadelphia. (**A**) Circuit and system components consisting of a pumpless, low-resistance oxygenator circuit, a closed fluid environment with continuous fluid exchange and an umbilical vascular interface. (**B**) Representative lamb cannulated at 107 days of gestation and on day 4 of support. (**C**) The same lamb on day 28 of support illustrating somatic growth and maturation. From Partridge et al (2017) under a CC-BY license.

The system incorporated a pumpless oxygenator circuit connected to the foetus of a lamb via an umbilical cord interface and a closed bag filled with artificial ‘amniotic fluid’ to reproduce the environment of the womb as closely as possible (Fig. 1). Lack of a pump avoided pulses of pressure that could possibly damage the lamb’s immature circulation.

The normal gestation period for sheep is about five months, just over half the human term. The authors showed that foetal lambs developmentally equivalent to extreme premature human infants 22 to 24 weeks old could be physiologically supported in this extrauterine device for up to 4 weeks, and even longer. The lambs maintained stable haemodynamics, with normal parameters for blood gas and oxygenation. Provided appropriate nutrients were supplied, lambs on the system exhibited normal growth of all systems including the brain, as well as lung maturation and myelination in the CNS, that is formation of the myelin sheath around nerves to ensure electrical conduction for signal transmission.

The authors recognized though that existing methods to insert an arterial cannula into the foetus as a substitute for the placenta would not work for humans, because umbilical arteries take a more tortuous course and the cannulas required would be too large. To prepare for possible future use in humans, they developed a new system comprising very short cannulas securely attached to the umbilical cord. This allowed a length of umbilical cord to run between the cannulas and the foetus, giving more freedom of movement and less likelihood of the connection being broken during the remaining extrauterine pregnancy. It also exploits the natural resistance of the umbilical cord to arterial occlusion events when there is a temporary reduction of blood supply.

## Discussing application in humans

Since then the CHOP group has been joined by others to evolve and refine AWT and perform further safety validation. A major focus has been testing various ways to connect the foetus to the oxygenation machine, since this is a critical stage that must be settled before human trials can begin. Groups in Spain, Japan, Australia, Singapore, the Netherlands, and another in the USA at the University of Michigan in Ann Arbor, have joined the effort.

These developments pushed the US Food and Drug Administration (FDA) to organise a seminar (Pediatric Advisory Committee Meeting Announcement - 09/19/2023|FDA) to discuss the safety and effectiveness of artificial womb technology, “including regulatory and ethical considerations for first-in-human studies.” At this stage, the FDA is interested solely in AWTs as alternatives to existing care protocols for extremely premature infants rather than early-stage interventions against congenital defects.

“These developments pushed the US Food and Drug Administration (FDA) to organise a seminar to discuss the safety and effectiveness of artificial womb technology…”

The seminar covered safety aspects and the motivation for the approach based on the lack of existing alternatives in ICUs. Mark Mercurio, Director of the Paediatric Ethics Program at Yale-New Haven Children’s Hospital and Director of the Program for Biomedical Ethics at Yale University School of Medicine, discussed the need for a new protocol for Caesarean delivery of the foetus mid-way through normal gestation when it otherwise would not previously have been permitted or clinically recommended. He pointed out that clarity over risks to the pregnant mother would be needed, as well as over the implications for her ability to conceive successfully in the future again. Mercurio also noted that the anticipated benefit to risk ratio must be superior to available alternatives.

However, another speaker, Peta-Maree Alexander, Director of the Boston Children’s Hospital ECMO (Extra Corporeal Membrane Oxygenation), hinted that alternatives were limited in efficacy because they expose the immature cardiovascular system to atmospheric oxygen too soon, negatively impacting myocardial development. The ultimate outcome is usually impaired diastolic and systolic cardiac function. ECMO, which is similar to the heart-lung by-pass machine used in open-heart surgery, is sometimes administered to premature babies to assist respiration, allowing hearts and lungs to rest.

The consensus emerging from this seminar and among those in the field is that firstly safety must be established as far as possible, and secondly that a sound ethical and legal framework must be developed, although the latter could vary between countries according to local laws and societal attitudes. “Any team that is taking this to clinical translation will need to demonstrate a good safety profile to transfer the foetus from intrauterine life onto the extrauterine system to ensure the best outcome for the neonate,” said Anna David, Professor and Consultant in Obstetrics and Maternal Foetal Medicine and Director of the Institute for Women’s Health at University College London Hospitals in the UK.

This transfer process involving connection to blood vessels in the umbilical cord is challenging, because the arteries are tiny and immediately start contracting as a baby is delivered. Surgeons will therefore have only minutes to complete this process, which as David pointed out must be done smoothly to avoid any damage to the baby’s immature cardio-vasculature. For this reason, Matthew Kemp, an obstetrician at the National University of Singapore whose team is also developing an AWT system, wants to see data on how the experimental animals delivered so far fare in the long term, followed by further study on non-human primates, before clinical trials should begin. That last step will itself raise ethical issues because of increasing societal concerns over experiments on our closest relatives.

## The road to the clinic

Meanwhile, there is groundswell building behind AWT research in the belief that clinical trials will begin at least within a few years. This has drawn in several institutions, such as the Barcelona Centre for Maternal-Fetal and Neonatal Medicine in Spain. That centre is investigating a similar system to CHOP’s in the belief that diverse efforts are required to bring AWT to the clinic. According to Eduard Gratacós, Director of the Institute Clinic for Gynaecology, Obstetrics & Gynaecology at the Barcelona Centre, clinical trials will have to begin at some stage and will inevitably elicit further issues that will have to be resolved. “We believe we could be ready for trials in 2 to 3 years,” he said. “The results of these first clinical trials and difficulties found will be paramount to understand if we are talking of something that could be a clinical reality in 5 to 10 years.”

“Meanwhile there is groundswell building behind AWT research in the belief that clinical trials will begin at least within a few years.”

Gratacós dismissed the argument that it would be better to focus research on minimising risk of preterm birth rather than spending a lot of money on developing ectogestation. “That’s all very well to say, but while of course we must strive to reduce these risks, it is very unlikely that we will completely eliminate the risks of extreme prematurity and severe complications such as severe foetal growth restriction or severe preeclampsia near viability,” he explained. “To say that an artificial womb/placenta will have marginal benefits is speculative. This is a formidable challenge for medicine, but once the technical limitations and difficulties—which we must acknowledge are huge—are overcome, it makes sense to believe that in the future a foetus surviving in ectogestation conditions would be better off than in a conventional ICU support as is done now.”

Gratacós is fairly sanguine over the technical challenges, arguing that the fundamental ones are mostly solved already. “We do need to refine our knowledge about certain aspects of cardiovascular physiology and foetal nutrition/growth,” he conceded. “However, at some point some of these challenges will not be perfectly solved in animal models, so we will then need to start clinical trials.” He further agreed that the first applications will be for treating premature births close to the earliest point of viability, that is around 22 to 24 weeks. “In a second stage it would certainly be logical to consider congenital malformations that need in utero therapy and could be treated in an artificial womb,” he added.

## Pre-natal therapies

That second stage will certainly take longer to arrive because it requires establishing protocols not just for the uterine transfer to the artificial womb but also for therapeutic manipulation within it with additional technical and safety-related challenges. Yet the motivation for such therapies is just as clear, as many of them have far greater prospects of success if conducted at an early developmental stage, as well as being easier to conduct and more affordable in some cases.

One study assessed the benefits of various interventions at the foetal stage, especially systemic gene therapy and identified three major advantages for administering gene therapies early on during the second trimester of pregnancy (Bose et al, 2021). The first is that small foetal size allows much lower dosing as a human foetus in the second trimester, the gestational age at which systemic gene therapy becomes technically feasible, is about 1% the size of a 1-year-old child.

Secondly, body compartments and cells are more accessible in the foetus. In particular, the foetal blood–brain barrier is still permeable so that systemic gene therapy can be applied more readily to central nervous system cell types. Moreover, progenitor cells are more prevalent in the foetus and often proliferative, which increases the probability of propagating a therapeutic correction or gene integration.

Thirdly, postnatal gene therapies can be impaired by immunologic responses to the transgenes and viral vectors to deliver them. It has already been demonstrated in animals that prenatal delivery circumvents immune resistance to viral transgenes, including Cas9 used for CRISPR gene editing.

While at least some gene therapies may be administered in utero, there are numerous congenital defects resulting from interactions between genetic and environmental factors that would have to be addressed prenatally in an artificial womb. Common defects include deformities like cleft palate and club foot that can be fixed postnatally. But there are also cardiac, neural tube and other defects that normally have lifelong consequences and would be best addressed at an early developmental stage before they cause collateral damage in other organs and tissue. According to the WHO (https://www.who.int/health-topics/congenital-anomalies#tab=tab_1), about 6% of babies worldwide are born with some congenital disorder, resulting in hundreds of thousands of associated deaths. The true number of cases may be much higher because statistics often do not include terminated pregnancies and stillbirths. It is quite possible then that AWT could eventually achieve even greater benefits in treating congenital defects than premature births.

“It is quite possible then that AWT could eventually achieve even greater benefits in treating congenital defects than premature births.”

## Ethical and legal issues

But apart from technical challenges there is also the ethical and legal dimension, which in its way is just as challenging, as highlighted by Elizabeth Chloe Romanis, Associate Professor in Biolaw at Durham Law School in the UK. “The technology raises a lot of very complex ethical issues, many or most of which are in need of examination ahead of first in human use,” she said. “These issues encompass a number of things, such as how we identify the right clinical population. And how do we obtain a proper informed consent? How do we ensure the pregnant person is centred in all of these decisions?”

Romanis also contends that a new definition is needed to describe AWT, and has even come up with a neologism herself. “I have argued that the entity in an artificial placenta is a wholly new entity—there has never been a human being gestating outside the human body before. I call it the gestateling, and I have recently explained why I think this is better than other terms people have suggested” (Romanis, 2023). “The debate about what the entity is and what we should call it is ongoing,” she continued. “I think that people often want to deny that the entity in an artificial placenta is unique because it raises all sorts of big questions like moral status. I think, however, that the unique status of the gestateling is not something that can or should be ignored.”

These questions are also being addressed by obstetricians interested in the ethical dimension, such as Joanne Verweij, Maternal-foetal Medicine Specialist at the Leiden University Medical Centre in the Netherlands. She noted that these issues will vary between countries and have yet to be thrashed out. “Patient identification and selection are critical as it should be safe and proportional both for pregnant women and child.”

There is also the question of cost, which is a major concern of Michael Harrison, a surgeon at the University of California, San Francisco, sometimes referred to as the father of foetal surgery because of his pioneering developments. “I am not involved with present programs, but think they are getting close,” he said. “I actually support the technology, just wonder about the cost.”

Thus, even if human clinical trials may occur within a few years, it is unclear when AWT will proceed to general use in the clinic. Costs and safety issues will likely be more critical than the bioethical and legal concerns, which are easier to resolve in countries that have a stronger focus on exploring new medical technologies to the benefit of patients.

### Supplementary information

Peer Review File

## Supplementary information

A peer review file is available at 10.1038/s44319-023-00022-4.
