# Supplementary material for: Exogestation for treating premature births and congenital diseases: Artificial womb technology edges towards first trials in humans
Source: EMBO Rep. 2024 Jan 5;25(1):17–20. doi: 10.1038/s44319-023-00022-4 (PMC10897319; doi:10.1038/s44319-023-00022-4)
Supplement: Supplementary file 1 — Peer Review File [file 44319_2023_22_MOESM1_ESM.pdf]

EMBO reports

Exogestation for treating premature births and congenital diseases

Philip Hunter
DOI: 10.15252/embr.202358477

Corresponding author(s): Philip Hunter (ph@philiphunter.com)

**Review Timeline:**	Submission Date:	13th Nov 23
	Accepted:	27th Nov 23

Editor: Holger Breithaupt

Transaction Report: This article was editorially reviewed.
